# Honorary Degrees

**Published:** 1892-07

**Authors:** 


### Editorial.

### HONORARY DEGREES.

In the earlier period of dental education, when the degree of Doctor of Dental Surgery was in its infancy, and its value was as yet an unsolved problem, the title was not only freely given, but it became an important unifying measure in the consolidation of professional interests.

The dental profession was confronted, at the organization of the earlier dental colleges, with a large body of men who had received their dental training from private dental offices and laboratories. This dead weight in opposition to progress was not only felt, but, in time, became obstructive, by its antagonistic position to all college work. The problem was one difficult of solution. To give these men all honorary degrees was out of the question; to confer a degree by examination was equally impossible, yet it was evident that dentistry could make but slow progress while this incubus remained.

One of the older colleges assumed the position that the latter course was the only creditable way out of the difficulty, and commenced the examination of men who had had fifteen years of actual practice. This was continued for some time, but the plan did not meet with approval of other schools, or generally in the profession, and was finally abandoned.

The issuing of spurious diplomas, and their extensive circulation throughout the world, brought, as is well known, all degrees of an honorary character into such disrepute that the majority of the colleges voluntarily refused to confer it, and the question was practically relegated to the slow changes which time would naturally effect.

The organization of the National Association of Dental Faculties very speedily brought this and related questions to an issue. It was clearly evident that this body would not favor degrees of the character described, and the matter was finally settled by a decisive vote refusing to allow a college to grant it upon one of the most distinguished of the older class of dentists. It was subsequently made impossible for any school associated with this organization to confer it. This action was universally accepted, not only as the proper course, but the only one possible for a dignified profession to pursue. Since that period the degree has ceased to appear at commencements.

It is with much regret, therefore, that we notice that this form of diploma has been revived in a neighboring State upon the organization of a new college, two gentlemen having received it, in order, it is presumed, to render them eligible to fill two of the chairs of the proposed institution. With their fitness for these positions we have nothing to do, but with the principle involved the whole profession is interested. The time is past for such a degree to be necessary. The training of men in the theoretical and practical branches has advanced so steadily with the lapse of years, that there should be no need to recur to an obsolete practice to secure the required ability to serve upon the staff of teachers.

Whatever may have been the value of this honorary title in the past, it has none now; indeed, it may be said to be an injury to the person possessing such a diploma; certainly it adds nothing to his position among his colleagues. The latter are more willing to accept an honest and intelligent evidence of correct practice than any sham presentation of ability however elegantly it may be inscribed.

The abuses of unearned degrees have multiplied to such an extent that it is the duty of all who value the good name of dentistry to set their faces in opposition to all attempts to return to a condition which had only expediency as an excuse for its adoption.

The degrees conferred were, doubtless, given under a wrong impression of the status of affairs, and with an idea of adopting a speedy way out of an embarrassing difficulty. Whatever may have been the motive, and we are not inclined to harshly judge it, the effect cannot be confined to a local area, but must have a widely-extended influence. If it will bring forcibly to the minds of all connected with educational matters that this degree can have no place in any well-regulated school; it will have a value not anticipated, probably, by those who conferred it.

It is gratifying to notice, as an indication of a healthful feeling, that the course of this university has provoked an extended comment of opposition. This has not arisen from any antagonism to a new school, but from a sincere conviction that a serious mistake has been made. It is to be hoped that it is not too late to correct it, for, in retracing the steps taken, the authorities of the university will show, in the best manner possible, that they have a respect for professional opinion, and have also a due regard for the future reputation of their new department.

The following quotations, taken from an article by a distinguished educator in dentistry, cover the whole ground, and should be seriously considered by those at the head of the new institution.

"The graduates of fifteen years ago would now scarcely recognize their Alma Mater. The granting of honorary degrees has been practically abolished. No college can confer a diploma except in course without first obtaining the consent of all the other schools in the association. Students must be in actual, daily, faithful attendance upon college lectures during the whole term of pupilage, or they are debarred from coming up for graduation. . . .

"This responsibility of each college to all the rest covers not only its manner of teaching, and of granting diplomas, but its discipline and internal regulation as well. At the last meeting of the Associated Faculties, one school was suspended from membership for a period of two years, because it had conferred a diploma upon a student who had not been faithful in a full attendance upon college lectures.[1] The suspension is a serious matter, for it means that no college will receive a student from it, or dismiss one to it, nor will its diploma be recognized in any manner. . . . A diploma should be a certificate of study, and it is a fraud when it represents anything else. . . . The whole system of conferring diplomas as marks of honor is wrong. . . . A college diploma is a certificate of study, or ought to be."

These views tersely expressed will certainly meet a sympathetic chord in every one opposed to any movement that implies retrogression, or an attempt to revive the practices of a dead past.

---

[1] This is not absolutely correct. The college in question matriculated two students long after the time limit.—Ed.

